# Current controversies with active surveillance management of small renal masses

**DOI:** 10.20517/2574-1225.2025.151

**Published:** 2026-05-12

**Authors:** Shane Kronstedt, Gal Saffati, Benjamin Yu, David E. Hinojosa-Gonzalez, David Dothan, Muammer Altok, Eric A. Singer, Eric C. Kauffman

**Affiliations:** 1 Scott Department of Urology, Baylor College of Medicine, Houston, TX 77030, USA; 2 Department of Urology, Roswell Park Comprehensive Cancer Center, Buffalo, NY 14263, USA; 3 Division of Urologic Oncology, The Ohio State University Comprehensive Cancer Center, Columbus, OH 43221, USA; 4 Department of Cancer Genetics and Genomics, Roswell Park Comprehensive Cancer Center, Buffalo, NY 14263, USA

**Keywords:** Small renal mass, active surveillance, progression criteria, delayed intervention, GLASS, consensus guidelines, renal cell carcinoma

## Abstract

Active surveillance (AS) management for patients with small renal masses (SRMs) is increasing globally, but questions remain regarding optimal AS practice. This review provides an evidence-based perspective on current controversies in SRM AS management. Considerable variation in AS utilization likely reflects non-standardization of patient selection criteria and differences among providers and healthcare settings. While most expert-consensus guidelines still restrict AS candidacy to patients with very small (< 1-2cm) renal tumors or significant health issues, increasing research supports AS to be an acceptable option for tumors up to 4 cm and a preferred option for many tumors up to 2 cm. For younger patients, AS appears oncologically safe but efficacy for long-term avoidance of delayed intervention (DI) remains unknown. Progression definitions for triggering DI still lack standardization, but there is general consensus for including thresholds based on some or all “GLASS” criteria [Growth rate; Longest tumor diameter; Adverse biopsy histology; Stage (≥ cT3a); Symptomatology]. SRM biopsy during AS can diagnose benign neoplasm with high accuracy to negate the need for DI, particularly when corroborated by computed tomography (CT) enhancement-based approaches such as tumor:cortex Peak Early Enhancement Ratio (PEER) scoring. In contrast, the value for biopsy in adverse histology detection remains more controversial. Advanced imaging modalities, including 99mTc-sestamibi single photon emission computed tomography (SPECT)/CT and [^89^Zr]Zr-girentuximab positron emission tomography (PET)/CT, may serve as useful adjuncts to biopsy during AS, while providing limited accuracy alone when biopsy is deferred. Future investigative efforts should focus on standardizing AS protocols, refining progression criteria for intervention, and addressing uncertainties about longer-term outcomes, particularly in younger patients.

## Graphical Abstract

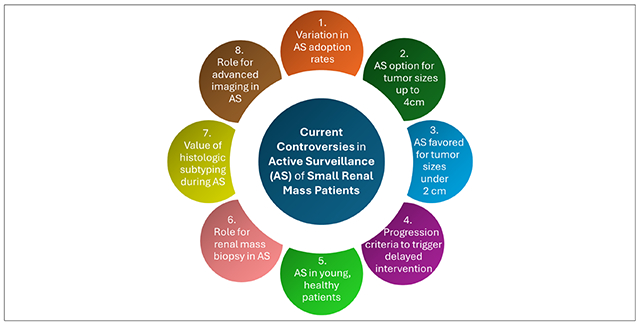

## INTRODUCTION

An estimated 81,610 new diagnoses and 14,390 deaths are expected from kidney cancer this year in the U.S., representing a significant disease burden despite modern advancements in detection and treatment^[1]^. Renal cell carcinoma (RCC) arising from the renal cortex is the most common kidney malignancy, accounting for approximately 90% of cases. RCC is itself heterogeneous, with most cases being the clear cell RCC subtype (70%-75%), followed by papillary (10%-15%), chromophobe (5%), and rare other subtypes^[2]^. Metastatic RCC is generally lethal; however, RCC that is clinically localized to the kidney is usually cured using extirpative therapy, with approximately one in four patients experiencing metastatic relapse. In addition to tumor stage and grade, a key prognostic factor for localized RCC patients is tumor size at diagnosis. Small renal masses (SRM, SRMs), defined as renal cortical tumors up to 4 cm, have an especially outstanding prognosis, due to their remarkably indolent behavior that includes a slow growth rate (GR), frequent growth cessation, and rare metastatic potential^[3–6]^. The rising incidence of RCC in recent decades is likely attributable to the increased incidental detection of smaller asymptomatic kidney tumors, resulting from high contemporary usage of cross-sectional imaging^[7]^. Accordingly, SRMs now make up approximately half of all new RCC diagnoses, and their overtreatment represents a significant healthcare challenge.

Given increased awareness of their clinical indolence, we have seen a change in the paradigm of how SRMs are managed over the past two decades, both in the U.S. and globally. Historically, surgical resection with partial or radical nephrectomy has provided the mainstay of treatment for patients with SRMs. However, as with any major operation, this surgery can incur significant morbidity, including long-term sequelae or even rare mortality, making it hard to justify in light of the non-aggressive nature of SRMs. While thermal ablation has emerged as a less invasive treatment option for SRMs, procedural morbidity still occurs, and oncologic control is inferior to resection; hence, surgery remains the current treatment of choice^[8–10]^. Given the clinical indolence of SRMs and potential morbidity of current treatment options, active surveillance (AS) has gained popularity as a first-line alternative to immediate treatment in select SRM patients^[3]^. In contrast to observation (i.e., watchful waiting), which defers curative treatment, AS management includes a plan upon tumor progression for delayed intervention (DI) with curative intent. However, many questions remain as to what constitutes optimal AS practice, particularly for patient selection, monitoring protocols, and the identity of progression triggers for DI. The current review provides an evidence-based perspective on these and other select controversies in contemporary AS management for SRM patients.

### WHY IS THERE SUCH A DISCREPANCY IN AS UTILIZATION AMONG UROLOGIST PRACTICES?

Despite increasing popularity, AS utilization in SRM patients still varies dramatically across practices. While only a minority of SRM patients (~10%-20%) undergo AS on a national level^[5,11]^, some academic U.S. centers are now utilizing AS even more often than immediate treatment. For example, per the 8-year experience reported out of Roswell Park Cancer Center, > 95% of all SRM patients were recommended and underwent AS, with immediate treatment reserved only for the rare patient subset with progression criteria already apparent at presentation^[12,13]^. Similarly, 61% of SRM patients in the multi-center Delayed Intervention and Surveillance for Small Renal Masses (DISSRM) registry have elected AS since 2009, including higher rates in more recent years^[4]^. Such variation is also prevalent within the community. Recent statewide population data from the Michigan Urological Surgery Improvement Collaborative (MUSIC) revealed stark differences in AS or observation adoption across more than a dozen academic or private urology practices, with some practices opting for AS/observation in more than 50% of SRM patients, compared to minimal AS/observation utilization (< 5%) for other practices^[14]^. While MUSIC oncologic outcomes await maturity, both the DISSRM consortium and Roswell Park have reported low metastasis rates of < 1% despite their high AS adoption^[4,13]^. Given this outstanding outcome, albeit from a limited number of centers, such dramatic variation in AS adoption raises critical questions about what drives AS patient selection and whether AS is being underutilized in specific settings^[15]^.

Factors related to the provider and healthcare setting likely contribute to this variability in practice patterns, which is unlikely to be explained by patient population differences alone. Differences in physician awareness or comfort with AS data may play a role, as some providers may be less familiar with recent literature supporting AS safety and efficacy, or may not fully trust these data without first wider adoption by peers or consensus guidelines groups^[16]^. Important in this regard may be the lack of detail and standardization across some consensus guidelines for AS patient selection. While major organizations endorse AS for SRM patients whenever the surgical risk outweighs oncologic risk, they do not always provide clarity (e.g., size criteria details) on when this threshold is met, allowing for variable interpretation. As an example, given that surgical mortality rates in general (~0.4%-0.8%)^[17,18]^ are higher than metachronous metastasis rates of SRMs < 3 cm (closer to 0.1% than 1%)^[6,19]^, the risk balance might be interpreted by some but not other providers to favor AS as the default approach for SRMs < 3 cm. In addition to the mortality risk, radical nephrectomy carries risks inherent to major surgery, including infection, hemorrhage, venous thromboembolism, and cardiopulmonary compromise, as well as acute and chronic renal failure and its long-term sequelae of anemia, osteoporosis and metabolic acidosis^[20]^. Partial nephrectomies share similar risks, in addition to other high-grade complications including pseudoaneurysm and urinary fistula^[18,21]^.

Disease uncertainty and lack of provider comfort can, in turn, compromise patient trust, which is critical for AS utilization^[22]^. In the Roswell Park study of near-universal recommendation of AS, even patients who did not follow up at Roswell Park after initial consultation still most often pursued AS, supporting the influence of the initial consultation experience^[12]^. However, the generalizability of this one high-volume AS practice remains unclear. In addition to the provider, factors related to the healthcare setting, including reputation and access to resources, may further affect patient trust and influence AS utilization. Although formal analyses are lacking, financial pressures might incentivize some provider recommendations for surgery over AS, including to retain patients in geographic regions with a high local density of competing urology practices. While the ethical implications warrant careful framing, they reflect real-world considerations influencing practice patterns^[23,24]^.

### CAN WE AGREE THAT AS SHOULD BE AN *OPTION* FOR ALL RENAL MASSES UP TO 4 CM IN SIZE?

Another controversial discussion point is whether we now have adequate data to consider AS for all SRMs up to 4 cm, regardless of patient health, rather than for only patients with tumors under 2 cm, poor health, or advanced age. Current consensus guidelines from the American Urological Association (AUA), National Comprehensive Cancer Network (NCCN), and American Society of Clinical Oncologists (ASCO) all endorse AS as an “option” for SRMs < 2 cm (AUA, NCCN) or < 1 cm (ASCO), but not for larger tumor sizes unless competing health risks exist [Table 1]^[25–27]^. It is our opinion that such restrictions may be overly conservative, since metachronous metastasis rates are negligible at SRM sizes up to 3 cm (and often lower than nomogram-personalized rates of surgical mortality), as underscored by only one case of AS metastasis at < 3 cm (2.8 cm) in a non-hereditary RCC patient reported through 2025 to our knowledge^[28]^, altogether supporting an increase in size cut-off up to at least 3 cm for AS consideration. Though more controversial, accumulating data also support AS as an option for the 3-4 cm size range of SRMs, based on outstanding metastasis-free survival and acceptable DI-free survival (> 50% at 5 years)^[4,12,13]^. In a 2012 systematic review of > 800 patients in the early AS literature, only two patients had a tumor size of 3-4 cm at the time of metastasis^[19]^. Among many more recent AS reports through 2025 totaling close to 2,000 patients, we are aware of only one additional documented case of metastasis that occurred during AS at a SRM size of 3-4 cm, although the precise denominator number of patients within this size range subset is unclear [Table 2]^[28–38]^. Consistent with the favorable oncologic risk in this greater size range, several consensus guideline groups including the International Consultation for Urological Diseases (ICUD) and Canadian Urological Association (CUA) have now endorsed AS as an option for all patients with SRMs up to 4 cm [Table 1]^[3,39,40]^. Nevertheless, given the low but non-negligible rate (~2%-3%) of metachronous metastasis for 3-4 cm tumors based on the surgical literature, closer monitoring and lower thresholds for DI should be considered for this higher-risk size range. Specific strategies for closer monitoring of tumors > 3 cm lack standardization. Reported approaches include a shorter-interval initial repeat scan (e.g., after three months before transitioning to every six months), a lower GR cut-off to trigger DI (e.g., > 3 mm/year instead of > 5 mm/year), and use of routine biopsy to assess for unfavorable histology or benign tumor histology^[3,12,13]^. Concerns that AS in patients with larger SRMs might preclude partial nephrectomy eligibility thus far appear to be unfounded, since AS series with size selection up to 4 cm report high rates of partial rather than radical nephrectomy at DI, similar to those rates reported in immediate surgery series^[12,31,35,41–44]^.

Additional tumor and patient factors that are independent of tumor size may further modify AS candidacy. For instance, patient comorbidity, tumor multifocality and cystic tumor nature each tend to make AS adoption more favorable. Another important consideration is the impact of patient anxiety on AS management, and *vice versa*. Mental health research using patient-reported outcomes (PROs) in contemporary AS cohorts is limited and heterogeneous, but suggests acceptable psychological tolerability.The DISSRM prospective observational comparative trial of AS *vs*. immediate intervention evaluated PROs as a secondary outcome measure and found no decline in mental health scores with AS management^[45,46]^, including no differences at all evaluated time points with the exception of significantly higher mental health scores for AS patients at 4 years^[45]^. More recently, Goldberg *et al*. also detected no differences in PRO-based mental health scores among 217 AS patients *vs*. 260 immediate intervention patients at predefined time points, although a minority subset (~15%) of AS patients with biopsy-confirmed malignancy had significantly lower scores^[47]^. While patient anxiety and other size-independent factors are clearly important modifying factors, we believe that a size range up to 4 cm provides a useful starting framework on which to consider AS candidacy.

### SHOULD AS BE *FAVORED* OVER IMMEDIATE TREATMENT FOR VERY SMALL RENAL MASSES (< 2 CM)?

A perhaps even more progressive question is whether AS should be the preferred management strategy over immediate treatment for all tumors < 2 cm. While CUA guidelines already advocate for AS as the standard-of-care preference for tumors < 2 cm^[39]^, other major consensus guidelines currently stop short of favoring AS over surgery at any tumor size unless a patient is unhealthy and/or old [Table 1]. There is, however, compelling rationale to favor AS management for < 2 cm renal masses based on both safety (oncologic) and efficacy (durable avoidance of treatment). Oncologic safety is supported by the virtual absence of metachronous metastasis and cancer-specific mortality at SRM sizes < 2 cm (< 0.1%)^[6]^ including no documented metastatic cases in the AS literature through 2025 to our knowledge, which suggests a lower AS mortality risk than that of rare surgical mortality. Secondly, a considerable portion (~1 in 3) of patients with a < 2 cm SRM have a benign neoplasm for which surgery is unnecessary; however, a diagnostic biopsy is technically challenging under 2 cm, and minor growth during AS can allow for a more reliable biopsy that may negate the need for surgery^[48]^. Finally, while 10-year data are still lacking, the vast majority (~90%) of SRMs < 2 cm do not require DI over at least five years, making longer-term avoidance of treatment a real possibility^[4,12,13]^. Although more research is clearly needed, it is not inconceivable that this argument could eventually be extended to even a < 3 cm cut-off, since the metastatic risk at this size range is similarly negligible and comparable to (often lower than) the mortality risk of surgery^[6,17–19]^. Beyond its mortality risk, surgery also has other morbidity risks described above, such as bleeding, urinary fistula, renal dysfunction, and metabolic dysfunction^[20,21]^.

### WHAT TRIGGERS SHOULD WE BE USING AS PROGRESSION CRITERIA FOR DI?

Defining clear and objective tumor progression criteria for conversion to DI during AS is essential to optimizing patient outcomes. However, the identity of these progression criteria lacks standardization and thus remains controversial, with most consensus guidelines not yet addressing this topic^[26,27,40,49]^. Currently, only the AUA, CUA and ICUD provide specific thresholds for defining progression during AS^[3,25,39]^. As with these guidelines, research on progression during AS has focused primarily on two major criteria for triggering intervention: size (longest tumor diameter, LTD) and GR^[3]^.

LTD is the best-established predictor of SRM metastasis risk, but there is no uniformly accepted standard for a specific size threshold to trigger DI during AS. The AUA guidelines currently condone a threshold of > 3 cm^[25]^. However, contemporary AS series have consistently described outstanding oncologic outcomes using a > 4 cm threshold, with only two cases of metastasis at a tumor size of 3-4 cm documented in the past decade to our knowledge [Table 2]^[4,12,13,35,50]^. Accordingly, other consensus guideline committees, namely the CUA and ICUD, instead endorse a larger size threshold of > 4 cm to trigger DI^[3,39]^.

GR is another progression criterion often used to trigger DI. Common consensus (including AUA, CUA and ICUD) favors a GR progression threshold of > 5 mm/year^[3,25,39]^, which is several fold faster than the typical SRM GR. GR is associated with adverse pathology and metastasis in many retrospective studies^[12,28,44,51–53]^. The vast majority (> 80%) of metastatic SRM AS cases with reported GR have grown faster than 5 mm/year^[19]^, compared to only a minority (~15%) of all SRM AS cases. Furthermore, all AS metastases with size < 4 cm and any GR reported through 2025 have had an above-average GR of > 3 mm/year [Table 2]^[19,28,50,54]^. Given some reported metastases with GR < 5 mm/year but not < 3 mm/year, the Roswell Park team has endorsed size-stratified GR thresholds to trigger DI whereby a > 5 mm/year GR threshold is used for tumors with LTD ≤ 3 cm, while a stricter threshold of > 3 mm/year GR threshold is used for tumors with LTD > 3 cm^[12,13]^.

Recently, the value of GR in defining progression has come under scrutiny. Using their multicenter prospective registry, DISSRM investigators reported that a rapid GR did not increase the risk of adverse pathology in their AS cohort^[4,55]^. Furthermore, they observed no cases of metastasis among a subset of patients who deferred DI despite a GR of > 5 mm/year^[56]^. However, the small size of this patient subset and expected rarity of metastasis among even higher risk SRM patients limits conclusions, and a prospective study that is designed and appropriately powered to assess the relationship between GR and metastasis is still needed. Nonetheless, these findings underscore the outstanding oncologic safety of AS for SRM patients regardless of tumor features, and the potential need to raise thresholds for triggering DI.

**Figure 1.**
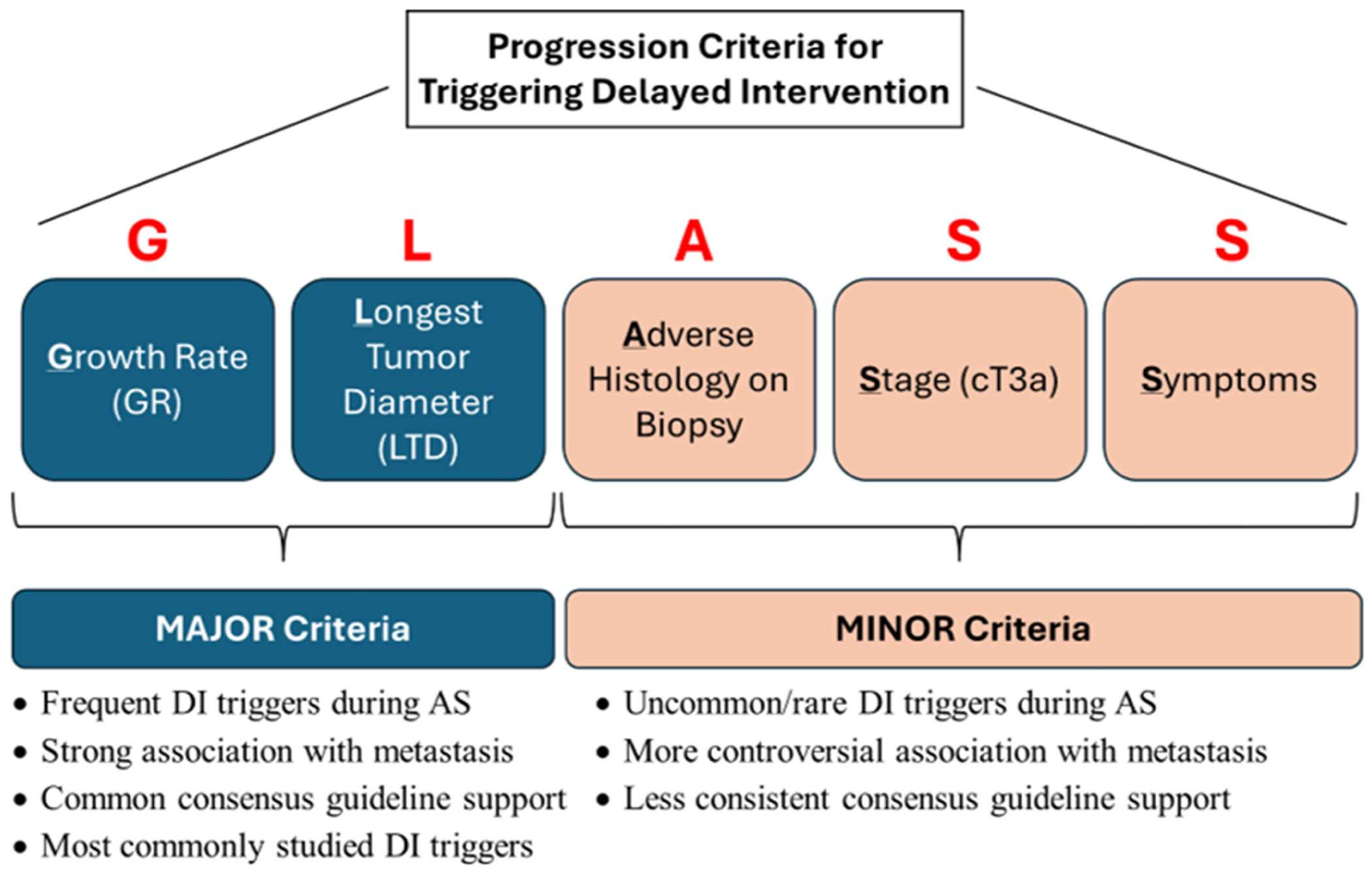
GLASS progression criteria for triggering DI in SRM patients on AS. DI: Delayed intervention; SRM: small renal mass; AS: active surveillance.

In addition to GR and LTD, other factors should trigger consideration of DI. These include unfavorable histology (e.g., high grade), invasive clinical tumor stage (i.e., cT3a), and symptom onset (e.g., hematuria or paraneoplastic effects). However, progression based on these thresholds is rare during AS, and hence they can be considered minor criteria^[12]^. The recently proposed GLASS criteria (Growth rate, Longest tumor diameter, Adverse biopsy histology, Stage, and Symptomatology) offer a structured framework integrating multiple oncologic risk factors to guide decision-making for DI *vs*. continued surveillance [Figure 1]^[3,12,13]^.

### WHAT IS THE ROLE OF AS IN YOUNGER PATIENTS WITHOUT MAJOR HEALTH ISSUES?

In contrast to its widespread acceptance for the elderly, the role of AS for younger and otherwise healthy SRM patients is a topic of growing controversy. A significant limitation of historical AS research has been its heavy selection bias towards elderly and/or unhealthy patients, and accordingly, expected rates of progression and delayed treatment have been unknown for younger patients who are also fit for surgery. Recently, two studies have shed some new light on the safety and utility of AS in younger patients. DISSRM investigators reported a subset analysis of 224 young patients in their prospective registry, including 68 AS patients. Although the follow-up duration for the AS patient subset was limited, no metastases were observed, and most young patients avoided DI, including a 5-year treatment-free survival rate approaching 70%^[57]^. More recently, the Roswell Park team reported the largest series to date of young and healthy AS patients (101 patients) and with relatively mature follow-up (median ~4 years). Their results mirror those of DISSRM, including a 68% 5-year DI-free survival and 100% metastasis-free survival^[37]^.

These oncologic outcomes, including the 0% rate of metastasis and just over 30% rate of DI at five years, appear comparable to most contemporary AS series enriched for elderly patients, which tend to report around a 0%-2% rate of metastasis and 20%-30% rate of DI over a similar time frame^[4,13,36,38,41]^. Younger age has been identified in several studies as a significant predictor for DI^[32,38,58]^, which can be expected since older patients more commonly defer DI (due to inadequate health or life expectancy) upon demonstrating progression. Thus, early research supports the oncologic safety of AS and its short-term utility for treatment avoidance in younger patients, but caution is warranted given the relatively small cohort sizes to date. Furthermore, decision-making considerations for younger AS candidates are distinct from their elderly counterparts in that they must take into greater account the lack of very long-term follow-up data (> 10 years), which leaves to question whether DI might become inevitable over more extended durations in an unacceptably high proportion of younger patients. Secondly, more consideration must be given to the cumulative psychological burden that might accompany AS management over such extended durations, even with stable disease. As a special consideration, younger patients have an increased lifetime risk of tumor multifocality^[59]^, and particularly when multifocality is already evident, AS may provide a benefit of delaying surgery until potential future *de novo* tumors can be resected simultaneously, thus limiting the lifetime number of surgeries. In summary, although AS appears to be safe in young and healthy patients with SRMs, its long-term utility in this patient subset remains unclear, and shared decision-making is warranted at this time.

### WHAT IS THE ROLE OF RENAL MASS BIOPSY DURING AS?

The role of renal mass biopsy in AS management remains an ongoing topic of controversy. Guidelines often recommend consideration of biopsy for patients to evaluate AS candidacy^[25,49]^, or more generally, whenever results would change management decisions^[27,39]^, yet its routine use is not universally adopted. For example, Roswell Park and the RCC Consortium of Canada perform biopsies in most AS patients^[12,60]^, in contrast to < 20% rates at many other high-volume AS centers^[4]^. The best example of where biopsy can alter management is with the diagnosis of benign tumor (not to be confused with a non-diagnostic biopsy that fails to identify tumor tissue), as this result can negate the need for intervention^[12]^. Most contemporary series indicate an accuracy of 100% or near-100% for distinguishing malignant *vs*. benign neoplastic histology using a needle-core biopsy^[61,62]^, reinforcing its utility in AS decision-making^[62]^. Furthermore, non-diagnostic rates in SRM patients are < 10%, with most cases converting to diagnostic after repeat attempts^[61]^. The Roswell Park series performed renal mass biopsy in over 60% of AS patients, with histology revealing 71% RCC, 23% benign, and 6% non-diagnostic. They demonstrated a 100% specificity and positive predictive value for malignancy on biopsy, while completely avoiding benign surgical resections at DI. These findings show how biopsy can be utilized with excellent accuracy to guide AS management decisions ^[12]^.

Additionally, the historical diagnostic challenge of differentiating benign renal oncocytoma from chromophobe RCC has been largely overcome with improved pathologist experience, as well as with specific radiographic enhancement measurement techniques such as the tumor:cortex Peak Early Enhancement Ratio (PEER) score. PEER scoring achieved 100% accuracy in prospectively differentiating these two subtypes, and its efficacy has been validated in multiple external reports^[63–65]^. PEER scoring is an attractive supplement to biopsy because it can be measured quickly by either urologists or radiologists using routine CT scans.

Another debated use of biopsy is the detection of unfavorable (adverse) histology as a progression criterion that may prompt intervention. However, although SRM biopsy has excellent specificity for high-grade RCC, tumor under-sampling and suboptimal sensitivity for high-grade foci within SRMs remain an ongoing challenge, limiting the reliability of biopsy for ensuring lack of progression during AS^[61]^. In contrast, given the well-established association between grade and metastatic risk, a high-grade biopsy result during AS is both reliable and useful, and should prompt careful consideration of intervention.

### IF A BIOPSY IS PERFORMED, DOES THE HISTOLOGIC SUBTYPE MATTER?

More recent AS research has raised the question of whether progression criteria should be tailored based on histologic subtype. Among the largest case series of biopsy-confirmed RCC cases on AS, Canadian researchers found that non-clear cell RCC tumors most often had no growth (median GR of 0 mm/year)^[35]^. Furthermore, the clear cell subtype had significantly faster growth and accounted for all metastatic cases during AS. Similarly, the Roswell Park team observed significantly worse progression-free survival for the clear cell subtype^[12]^. Slower growth and scarcity of metastases raise the possibility that more lenient AS triggers (e.g., size > 5 cm or GR > 8 mm/year) might be appropriate for non-clear cell RCC subtypes, although more study is needed. While surgical literature is conflicting as to whether histologic subtyping has prognostic value independent of other prognostic pathological variables, including grade and T stage^[66]^, these other pathological variables are not reliably assessed during AS. Therefore, histologic subtyping may be more valuable in the AS setting than the surgical setting. It is thus reasonable to expect histologic subtyping to become an important element of AS research in the near future.

### IS THERE A ROLE FOR ADVANCED IMAGING MODALITIES IN DIFFERENTIATING BENIGN FROM MALIGNANT SRMS?

Current protocols for radiographic monitoring during AS include conventional imaging modalities of computed tomography (CT), magnetic resonance imaging (MRI), and ultrasound. Cross-sectional imaging with either CT or MRI is generally used initially, while ultrasound is preferred for longer-term surveillance after tumor stability is established (i.e., consistent slow or no growth)^[3,12]^. Multiple newer emerging imaging modalities have shown exciting promise as an adjunct to conventional imaging in select scenarios, particularly for assisting with histologic diagnosis to better risk-stratify patients for AS or treatment. One such modality is [^89^Zr]Zr-girentuximab positron emission tomography (PET)/CT, which aims to non-invasively detect clear cell RCC using a monoclonal antibody against carbonic anhydrase IX (CAIX), a protein commonly expressed in clear cell RCC but not other histologic subtypes. This modality was recently evaluated in a phase 3 prospective trial (ZIRCON), which revealed an impressive sensitivity of 85.5% and specificity of 87.0% for clear cell RCC detection^[67]^. Another advanced imaging modality, 99mTc-sestamibi single photon emission computed tomography (SPECT)/CT, has been investigated for its ability to non-invasively differentiate benign renal oncocytic tumors from malignant renal tumors. This modality utilizes radiotracer uptake to identify tissue that is rich in mitochondria, given that benign tumors are generally mitochondrial rich while malignant tumors are commonly mitochondrial poor. Two prospective studies have supported good diagnostic accuracy of 99mTc-sestamibi SPECT/CT for detecting benign tumors, including sensitivity and specificity of 80%-90%. Still, both were limited by a very small number of benign cases (*n* = 6 or 7)^[68,69]^. A challenge with this approach has been false positives in oncocytic/eosinophilic variant subsets of RCC with high mitochondrial content, particularly the eosinophilic variant of chromophobe RCC. In the largest series of chromophobe RCC patients evaluated with 99mTc-sestamibi SPECT/CT, 50% (9/18) of cases had false positivity^[70]^. In summary, both [^89^Zr]Zr-girentuximab PET/CT and 99mTc-sestamibi SPECT/CT appear promising in their diagnostic accuracy and potential to risk-stratify patients, offering non-invasive strategies for SRM patients who are considering AS^[71]^. While accuracy and depth of histologic subtyping appear inadequate to replace biopsy altogether, both modalities may have a role as an adjunct to biopsy (e.g., when non-diagnostic or non-definitive) or as a stand-alone test for select SRM patients who prefer to avoid biopsy.

## CONCLUSIONS AND FUTURE DIRECTIONS

AS is a validated approach to SRM management, yet its implementation remains highly variable, and many practices still underutilize this strategy. While provider familiarity, competitive practice environments, and non-standardized guidelines may contribute to these discrepancies, emerging evidence supports a need for broader adoption of AS. Future investigative efforts should focus on standardizing AS protocols, refining progression criteria for intervention, and addressing lingering uncertainties about long-term outcomes, particularly in younger patients. Also requiring more study are financial and healthcare system barriers to AS adoption, mental health repercussions, and global utilization including outcomes outside of Europe and North America. Finally, emerging biomarkers and radiomics are eagerly awaited and hold great promise to improve AS patient care. In the interim, AS will likely continue to expand as a cornerstone of SRM management, maintaining oncologic safety while minimizing overtreatment as our understanding evolves.

## Figures and Tables

**Table 1.** Summary of current expert-consensus guidelines for AS patient selection

Guidelines Committee	Date	Selection criteria for AS		
		Option/may be offered	Preferred/should be offered	
AUA^[25]^	2021	XXX	XXX	Tumor size < 2 cm, or predominantly cystic masses (cyst size not specified) Any renal mass if treatment risks of death outweigh the oncologic benefit
CUA^[39]^	2022	XXX	XXX	Tumor size 2-4 cm Tumor size < 2 cm
EAU^[49]^	2024		XXX	Tumor size ≤ 4 cm if a patient is frail and/or comorbid
ICUD^[3]^	2021	XXX	XXX	Tumor size ≤ 4 cm Any renal mass if treatment risks outweigh the oncologic benefit
ASCO^[27]^	2025	XXX	XXX	Tumor size ≤ 4 cm if significant comorbidities or limited life expectancy (< 5 years) Tumor size <1 cm
ESMO^[40]^	2024	XXX		Tumor size ≤ 4 cm especially in a patient with short life expectancy
NCCN^[26]^	2025	XXX		Tumor size < 2 cm, or ≤ 4 cm if predominantly cystic, or any cT1 patients with significant risks of death or morbidity from intervention

AS: Active surveillance; AUA: American Urological Association; CUA: Canadian Urological Association; EAU: European Association of Urologists; ICUD: International Consultation for Urological Diseases; ASCO: American Society of Clinical Oncologists; ESMO: European Society for Medical Oncology; NCCN: National Comprehensive Cancer Network.

**Table 2.** Incidence, size and GR of metastatic SRMs during AS reported in the last decade (2015-2025)

Study (first author, year)	Institute, location	Patients (*N*)	SRM size (median), cm	Follow up duration (median), months	Metastasis during AS (*n*, %)	Size of each mSRM at AS initiation, cm	Size of each mSRM at metastasis, cm	GR of each mSRM, mm/year
Zhang 2015^[28]^	Peking University, China	60	1.9	27	6 (10)	1.6	4.4	2
						1.9	2.8	6
						0.1	8.0	47
						3.6	7.0	13

Schiavina 2015^[29]^	University of Bologna, Italy	70	2.7	61	2 (2.9)	NR	NR	NR

Celtik 2017^[30]^	Northwell Health, USA	89	2.4	29.9	3 (3.3)	2.0 (median)	4.3	9
							4.9	13^†^
							6.15	17^†^

Paterson 2017^[31]^	TUCAN, United Kingdom	158	2.2	18.9 (cystic) 19.5 (solid)	7 (4.4)	3.7	8.1	21
						2.8	5.7	8
						4.0	NR	NR
						1.3	4.6	6
						4.0	10.0	11
						1.7	3.6	6
						3.0	4.0	7

McIntosh 2018^[32]^	Fox Chase Cancer Center, USA	457	2.1	67	8 (1.8)	2.2	NR	7 (median)

Petros 2019^[33]^	MD Anderson Cancer Center, USA	272	1.7	58	4 (1.5)	NR	NR	NR

Whelan 2019^[34]^	Dalhousie University, Canada	103	2.1	55.5	2 (1.9)	3.7	4.8	4
						4.6	8.5	6

Finelli 2020^[35]^	RCCC;	136*	2.5 (RCCC) 2.0 (PMCC)	31.2 (RCCC) 46.8 (PMCC)	6 (4.4)*	NR	NR	NR

Bertelli 2021^[36]^	Careggi University, Italy	158	1.6	25	0 (0)	NA	NA	NA

Altok 2023^[13]^ (updated from Menon 2021^[11]^)	Roswell Park Comprehensive Cancer Center, USA	201	2.0	47	0 (0)	NA	NA	NA

Alkhatib 2025^[4]^	DISSRM consortium, USA	581	1.7	38 (without DI) 40 (with DI)	3 (0.5)	NR	NR	NR

Ajami 2025^[38]^	Multicenter, Spain	384	2.05	43 (mean)	0 (0)	NA	NA	NA

† GR shown does not necessarily correspond to the same row for column “Size of each mSRM at metastasis, cm”.

* Study included only biopsy-confirmed RCC cases.

GR: Growth rate; SRMs: small renal masses; AS: active surveillance; mSRM: metastatic small renal mass; NR: not reported; TUCAN: Tayside Urological Cancers Network Database; RCCC: Renal Cell Carcinoma Consortium of Canada; PMCC: Princess Margaret Cancer Center; NA: not applicable; DISSRM: Delayed Intervention and Surveillance for Small Renal Masses; DI: delayed intervention; RCC: renal cell carcinoma.

## Data Availability

Not applicable.
